# Erratum: Therapeutic Atmosphere in Psychotherapy Sessions. *Int. J. Environ. Res. Public Health* 2020, *17*, 4105

**DOI:** 10.3390/ijerph17145241

**Published:** 2020-07-20

**Authors:** Marte L. Siegel, Eva M. Gullestad Binder, Hanne Sofie J. Dahl, Nikolai O. Czajkowski, Kenneth L. Critchfield, Per A. Høglend, Randi Ulberg

**Affiliations:** 1 Department of Psychology, University of Oslo, Forskningsveien 3A, 0373 Oslo, Norway; h.s.j.dahl@psykologi.uio.no (H.S.J.D.); n.o.czajkowski@psykologi.uio.no (N.O.C.); 2 Vestfold Hospital Trust, Halfdan Wilhelmsens alle 17, 3103 Tønsberg, Norway; 3 James Madison University, Department of Graduate Psychology, Harrisonburg, VA 22807, USA; critchkl@jmu.edu; 4 Division of Mental Health and Addiction, University of Oslo, Kirkeveien 166, 0450 Oslo, Norway; p.a.hoglend@medisin.uio.no (P.A.H.); randi.ulberg@medisin.uio.no (R.U.); 5 Department of Psychiatry, Diakonhjemmet Hospital, Forskningsveien 7, 0370 Oslo, Norway

The authors wish to make the following correction to their paper [1]. Marte L. Siegel and Eva M. Gullestad Binder made equal contributions to the manuscript. The authors, therefore, wish to replace:

Marte L. Siegel ^1,^*, Eva M. Gullestad Binder ^1,^*, Hanne Sofie J. Dahl ^1,2^, Nikolai O. Czajkowski ^1^, Kenneth L. Critchfield ^3^, Per A. Høglend ^4^ and Randi Ulberg ^4,5^

^1^ Department of Psychology, University of Oslo, Forskningsveien 3A, 0373 Oslo, Norway; h.s.j.dahl@psykologi.uio.no (H.S.J.D.); n.o.czajkowski@psykologi.uio.no (N.O.C.)

^2^ Vestfold Hospital Trust, Halfdan Wilhelmsens alle 17, 3103 Tønsberg, Norway

^3^ James Madison University, Department of Graduate Psychology, Harrisonburg, VA 22807, USA; critchkl@jmu.edu 

^4^ Division of Mental Health and Addiction, University of Oslo, Kirkeveien 166, 0450 Oslo, Norway; p.a.hoglend@medisin.uio.no (P.A.H.); randi.ulberg@medisin.uio.no (R.U.)

^5^ Department of Psychiatry, Diakonhjemmet Hospital, Forskningsveien 7, 0370 Oslo, Norway

* Correspondence: marte.siegel@gmail.com (M.L.S.); evamgbinder@gmail.com (E.M.G.B.)

with

Marte L. Siegel ^1,^* ^,†^, Eva M. Gullestad Binder ^1,^*^,†^, Hanne Sofie J. Dahl ^1,2^, Nikolai O. Czajkowski ^1^, Kenneth L. Critchfield ^3^, Per A. Høglend ^4^ and Randi Ulberg ^4,5^

^1^ Department of Psychology, University of Oslo, Forskningsveien 3A, 0373 Oslo, Norway; h.s.j.dahl@psykologi.uio.no (H.S.J.D.); n.o.czajkowski@psykologi.uio.no (N.O.C.)

^2^ Vestfold Hospital Trust, Halfdan Wilhelmsens alle 17, 3103 Tønsberg, Norway

^3^ James Madison University, Department of Graduate Psychology, Harrisonburg, VA 22807, USA; critchkl@jmu.edu 

^4^ Division of Mental Health and Addiction, University of Oslo, Kirkeveien 166, 0450 Oslo, Norway; p.a.hoglend@medisin.uio.no (P.A.H.); randi.ulberg@medisin.uio.no (R.U.)

^5^ Department of Psychiatry, Diakonhjemmet Hospital, Forskningsveien 7, 0370 Oslo, Norway

* Correspondence: marte.siegel@gmail.com (M.L.S.); evamgbinder@gmail.com (E.M.G.B.)

^†^ These authors contributed equally to this work.
